# The impact of diel vertical migration on fatty acid patterns and allocation in *Daphnia magna*

**DOI:** 10.7717/peerj.8809

**Published:** 2020-04-17

**Authors:** Meike Anika Hahn, Eric Von Elert

**Affiliations:** Department of Biology, University of Cologne, Cologne, North Rhine-Westphalia, Germany

**Keywords:** *Daphnia*, Diel vertical migration, Kairomone, Fish, Zooplankton, Fatty acid

## Abstract

In freshwater zooplankton diel vertical migration (DVM) is a widespread predator-avoidance behavior that is induced by kairomones released from fish. Thereby zooplankton reduces predation by fish by staying in deep and dark colder strata during daytime and migrating into warmer layers during night, and thus experiences diel alterations in temperature. Constantly lower temperatures have been shown to increase the relative abundance of polyunsaturated fatty acids (PUFAs) in *Daphnia* sp. Furthermore, a low dietary supply of the ω3-PUFA eicosapentaenoic acid (EPA) has been shown to limit the induction of DVM in *Daphnia magna* and the performance of *D. magna* under fluctuating temperatures, as experienced during DVM. In nature DVM of *D. magna* in response to fish is accompanied by the presence of fish-borne kairomone and diel fluctuations of depth dependent-parameters like temperature, food, and oxygen supply. Here we investigated the effect of factors, which are differing between *Daphnia* that perform DVM and those which do not. We selected to examine the effect of changing temperature and light conditions and of the presence/absence of fish kairomones on *D. magna.* For this purpose, we conducted a full factorial experimental design in which we grew *D. magna* under constantly warm temperatures in a diel light-dark regime or under alternating temperatures in darkness crossed with the presence or absence of fish kairomones. We analyzed the fatty acid composition of mature animals and of their offspring in each treatment. Simulation of the light and temperature regime of migrating animals in presence of the fish kairomone resulted in an increased relative allocation of the ω3-PUFA EPA, from adult animals to their offspring, manifesting as decreased EPA concentrations in mothers and increased EPA concentrations in their offspring in response to simulated DVM (mothers). Additionally, EPA concentrations in the offspring were affected by the interaction of simulated DVM and the fish cue. The presence of the fish kairomone alone increased the EPA concentration in the offspring, that was not experiencing simulated DVM. These findings lead to the conclusion that the temperature and light regime associated with DVM alone, as well as in combination with the DVM-inducing fish kairomones, alter the allocation of fatty acids to the offspring in a manner, which is beneficial for the offspring under the decreased average temperatures, which migrating animals are exposed to. A low dietary supply of ω3-PUFAs may constrain *D. magna*’s amplitude of DVM, but our results suggest that the next generation of animals may be capable of regaining the full DVM amplitude due to the effect of the fish kairomone and the experienced temperature fluctuations (and darkness) on tissue fatty acid composition. These findings suggest that fatty acid limitation in DVM performing *Daphnia* may be more severe for the maternal than for the offspring generation.

## Introduction

In lakes and ponds predation by fish is recognized as an important selective force which has been shown to shape trophic cascades and to affect numerous aspects of ecosystem ecology (Gliwicz, 1986; Reichwaldt & Stibor, 2005). This holds in particular for predation by planktivorous fish, which has led to the evolution of defenses against predation by fish that comprise changes in behavior, life-history and phenotype in freshwater zooplankton (Lampert, 1993; Weider & Pijanowska, 1993; Tollrian, 1994; Von Elert, 2012).

Diel vertical migration (DVM) of zooplankton is a widespread predator avoidance behavior in aquatic ecosystems (Gliwicz, 1986; Lampert, 1989; Hays, 2003). The risk of daytime predation by fish is reduced when zooplankton (e.g., *Daphnia magna*) prey resides in the deep, dark and colder layer of the water column. At night, *D. magna* migrates into the warmer, upper water stratum to minimize demographic costs associated with spending time in the cold, food-depleted deep-water refuge (Loose & Dawidowicz, 1994). The onset of DVM requires kairomones, i.e., chemicals released by fish (Dodson, 1988; Hahn et al., 2019) causing negative phototaxis (Van Gool & Ringelberg, 2002), so that DVM-performing *Daphnia* sp. are residing in darkness.

*Daphnia* represent a keystone genus in lakes and ponds, as they control the abundance of primary producers and as they represent a major food source for higher trophic levels (e.g., fish) in pelagic food webs (Hays, 2003; Haupt et al., 2009). DVM of *Daphnia* sp., therefore, has ecosystem-wide consequences on phytoplankton dynamics, nutrient recycling, and the vertical transport of matter (Reichwaldt & Stibor, 2005; Haupt et al., 2009).

Crustaceans are incapable of synthesizing long-chain (20-22 carbon atoms) polyunsaturated fatty acids (PUFAs) de novo and therefore require a dietary source of these lipids to satisfy their physiological demands (Ahlgren et al., 1990). In PUFAs several major classes are distinguished by the position of the first double bond, when counted from the terminal methyl group. In ω3-PUFAs, this double bond is at the third position from the terminal carbon atom (methyl end). In ω6-PUFAs it is at the sixth position. PUFAs cannot be converted between these two classes (Weers, Siewersen & Gulati, 1997) . However, within each class, elongation and desaturation of PUFAs is possible, although in several cases the rates have been shown to be too low to meet demands (Von Elert, 2002; Taipale, Kainz & Brett, 2011). Accordingly, the content of particular PUFAs in natural phytoplankton has proven to be a powerful predictor for *Daphnia* growth (Müller-Navarra et al., 2000; Wacker & Von Elert, 2001), which strongly suggests that *Daphnia* populations are seasonally or at least occasionally under bottom-up limitation by a low PUFA content in their natural diet (Hartwich et al., 2012). Supplementation experiments have demonstrated that a low content of PUFAs in their diet may constrain *Daphnia’s* growth on algae (Von Elert, 2002) and on cyanobacteria after supplementation with sterols (Von Elert, Martin-Creuzburg & Le Coz, 2003). Reproduction has as well been shown to be limited by a cyanobacterial diet due to the lack of PUFAs and sterols (Martin-Creuzburg & Von Elert, 2009). Conclusively, a co-limitation of *Daphnia* growth and reproduction by sterols and PUFAs has been proven (Martin-Creuzburg, Sperfeld & Wacker, 2009). Furthermore, a low content of the ω3-PUFA EPA in phytoplankton may constrain DVM in *D. magna*, which suggests that DVM-performing *D. magna* may have an increased EPA requirement (Brzezinski & Von Elert, 2015).

In a stratified lake *Daphnia* are exposed to diel changes in temperature (e.g., 12 °C change) (Loose & Dawidowicz, 1994) during DVM, which results in considerably lower mean temperatures (Stich & Lampert, 1981). For *D. magna* it has been shown that decreased temperature leads to an increased tissue concentration of the sum of PUFAs, suggesting a higher PUFA requirement at decreased temperatures (Sperfeld & Wacker, 2012; Von Elert & Fink, 2018). These changes are in accordance with homeoviscous adaptation of poikilotherms to lower temperatures, which try to maintain membrane fluidity constant through changes in membrane lipid composition (Hazel, 1995). This so-called homeoviscous adaptation of membranes, (Hazel & Williams, 1990) postulates that the most common adaptation of poikiloherms towards decreasing temperatures is to decrease the concentration of saturated fatty acids (SFAs) in cell membranes, which decreases the threshold ambient temperature at which the transition of membranes to the solid phase occurs. In other words, a decrease of SFAs increases membrane fluidity (Hazel & Williams, 1990; Hazel, 1995; Guschina & Harwood, 2006). Upon exposure to constantly low temperatures increases in PUFA content were observed in *D. magna* (Sperfeld & Wacker, 2012; Von Elert & Fink, 2018), supporting the hypothesis of homeoviscous adaptation. Furthermore, limitation of *D. magna* growth and reproduction by the well-studied ω3-PUFA eicosapentaenoic acid (EPA) becomes even more pronounced under decreasing temperatures (Sperfeld & Wacker, 2012). These findings point at homeoviscous adaptation in membranes of poikilotherms in response to constantly low temperatures and raise the question, how DVM-performing *Daphnia* sp., that are thus exposed to profoundly fluctuating temperatures, cope with these temperature changes with respect to their fatty acid composition.

DVM is clearly distinguished from experimental exposure of *Daphnia* sp. to constantly lower temperatures by the fact that DVM-performing animals are exposed to diel alterations of several factors like temperature, food availability, oxygen levels, darkness and to the fish kairomone. Considering that a low availability of the ω3-PUFA EPA restricts DVM in *D.magna* (Brzezinski & Von Elert, 2015), strengthens the following assumption: We hypothesize that DVM leads to increases of the UFA/SFA ratio of maternal *D. magna* as an adaptive response to decreased experienced average temperatures. In a study investigating the effects of temperature fluctuations and EPA supply on *Daphnia* fitness and fatty acid composition, this effect was not shown (Isanta Navarro et al., 2019). Nevertheless, we expect that a bioassay allowing for longer acclimation of the animals to the fluctuating temperatures, should reveal an increased UFA/SFA ratio. We assume that such an increase in the UFA/SFA ratio is adaptive and therefore expect to see this in the offspring generation of migrating animals, which would contradict the finding that under constantly low temperatures, no effect on the fatty acid composition of *D. magna* eggs was shown (Sperfeld & Wacker, 2012). Recent results on the effect of EPA supplementation on *D. magna* suggest that the ability of animals to cope with temperature fluctuations experienced during DVM, increases with EPA supply, as could be demonstrated for growth and reproduction (Isanta Navarro et al., 2019). Due to the DVM limiting character of the ω3-PUFA EPA (Brzezinski & Von Elert, 2015), we further hypothesize that EPA concentrations in mothers and their offspring increase under simulated DVM. In order to differentiate the overall effect of DVM from that of the kairomone only, we quantified fatty acids in *D. magna* exposed to constantly warm temperatures or to alterations of temperature and darkness (the latter will be referred to as simulated DVM) crossed with the absence/presence of fish kairomones.

## Material and Methods

### Animal and algal cultures

*Daphnia magna* clone B, originating from Grosser Binnensee Germany (Lampert, 1991), has frequently been used for bioassays investigating diel vertical migration (Brzezinski & Von Elert, 2015; Hahn et al., 2019; Isanta Navarro et al., 2019). Synchronized cohorts of max. 15 *D. magna* were cultivated in 0.8 L of filtered (0.45-µm filter) and aged (four days) tap water at 20 °C under dim light conditions. The animals were fed every other day with 2 mg C/L of *Cryptomonas* sp. (strain SAG 26.80 Culture Collection of Algae at the University of Goettingen (SAG), Germany). *Cryptomonas* sp. was grown in 5 L semicontinuous batch cultures by replacing 20% of the culture with fresh, sterile Cyano medium (Von Elert & Jüttner, 1997) with vitamins (thiamine hydrochloride 300 nM, biotin 2 nM, and cyanocobalamine–vitamin B12 0.4 nM) every other day.

### Life history experiment

A full factorial life history experiment investigating the factors “simulated DVM” and “fish cue” was conducted using the test animal *D. magna*. The bioassay was initiated with synchronized neonates of the 3rd clutch that were not older than 24 h. During the experiment seven animals were kept in 300 mL filtered and aged tap water (see above) containing 2 mg C/L of *Cryptomonas* sp. Volumes of the algal food suspension corresponding to 2 mg C/L were supplemented every day, while the animals were transferred into fresh media every other day until the first clutch was released. It took 6–8 days in the treatments without simulated DVM and 14–18 days in those, were DVM was simulated.

Crossing the experimental factors “simulated DVM” and “fish cue” resulted in four treatments. Every treatment was replicated four times. In treatments exposed to “simulated DVM” the animals were kept in darkness and experienced temperature alterations comparable to those experienced during DVM (Stich & Lampert, 1984; Mikulski et al., 2017; Isanta Navarro et al., 2019). The jars containing the animals were therefore placed in a dark water bath with alternating temperatures, where 8 °C simulated residence of the animals in the hypolimnion of a lake and 21 °C simulated residence in the epilimnion (Fig. S1). The absence of DVM was simulated by incubating the experimental jars in a water bath at constant warm temperatures (21 °C) and application of a 16:8 h light-dark cycle (light: 11 ± 1 µmol m^−2^ s^−1^), corresponding to temperature and light conditions experienced by animals residing in the epilimnion of a stratified lake (Lampert & Sommer, 2007).

The presence of fish, was mimicked by supplementing the media with an extract of fish incubation water of *Rutilus rutilus* that has earlier been shown to contain all DVM-inducing kairomone (Von Elert & Loose, 1996; Hahn et al., 2019). Six pre-starved (24 h) *R. rutilus* (body size 10–20 cm) were incubated in 16 L of tap water at 18 °C for 24 h. The water was filtered <0.45 µm, and the kairomone was extracted from the water by C_18_ solid-phase extraction (SPE) (Mega Bond Elut, C_18_-bonded silica, mass: 75 g, Agilent Technologies) as according to Von Elert & Loose (1996). Briefly, 4 L of the incubation water were adjusted to 1% methanol and passed through the cartridge. After a washing step with 1% methanol, the cartridge was eluted with 200 mL of methanol, and this eluate was evaporated to dryness and re-dissolved in methanol to yield 80 µL of the fish incubation water extract. The extract will from now on be called the fish cue, since it is not a purified kairomone. The fish cue was added to the respective treatments at concentrations that corresponded to fish densities of three fish in eight liters of non-processed fish incubation water. The negative control, simulating the absence of fish, was prepared by exposing the experimental animals to the same volume of pure methanol instead of the fish cue.

### Life history parameters

The somatic growth rate (g) was calculated according to the formula: g = (ln Wt − ln W_0_) × t^−1^, where W_0_ is the initial dry mass of neonates, W_t_ is the dry mass of the individual at the end of experiment, and t is the duration of the experiment (Wacker & Von Elert, 2001). At the beginning of the experiment, two times eight neonates were removed for the determination of W_0_. When experimental animals had released the first clutch, one or two adult *D. magna* and 10 neonates of each replicate were removed for the determination of *W*_*t*_ and of individual offspring mass. Dry mass was determined after animals had been dried at 60 °C over night.

Size at first reproduction was determined by taking digital photos of experimental animals, which carried the first clutch in their brood pouch, through a dissecting microscope set to a magnitude of 65 and subsequent measurement of their body lengths from these pictures using the software ImageJ (1.51k, USA). The length between the upper rim of the complex eye and the base of the tail spine was measured. The dissecting microscopy also allowed for counting of the clutch sizes.

### Analysis of fatty acids

For the quantification of fatty acids either eight neonates from the start of the experiment, five adult individuals or ten neonates from the first offspring generation were used. Exceptions concerned two replicates of the treatment simulated DVM x no fish cue, for which only one and four adult *D. magna* were analyzed. Depending on the dry mass of the animals in the sample, between 2 and 25 µg tricosanoic acid methyl ester (C23:0 ME) in isohexane were added as internal standard after the addition of 5 mL of dichlormethane/methanol (2:1, v:v) and storage at −20 °C over night. Cells were disrupted by vortexing and sonication for 1 min to de-liberate lipids. After a centrifugation step at 3200 g for 5 min the supernatant was transferred into a new reagent tube. The remaining tissue was extracted again in the same way using 3 mL of dichlormethane/methanol (2:1, v:v). Both extracts were pooled and the extraction solvent was evaporated to dryness under a nitrogen stream at 40 °C. Fatty acids were then transesterified into fatty acid methyl esters (FAMEs) by addition of 5 mL of 3 N methanolic HCl and incubation at 70 °C for 20 min. The FAMEs were extracted twice by adding 2 mL of isohexane, vortexing for 30 s and subsequent collection and pooling of the isohexane phases. Pooled extracts were evaporated to dryness under a nitrogen stream at 40 °C and re-dissolved by twice adding 100 µL of isohexane. The obtained 200 µL of extract were transferred into vials, evaporated to dryness again and finally dissolved in 50 µL of isohexane, from which 1 µL was used for the quantification of FAMEs.

FAMEs were separated by gas chromatography on a 6890-N GC system (Agilent Technologies, Waldbronn, Germany) equipped with a DB-225 capillary column (30 m, 0.25 mm i.d., 0.25 µm film thickness, J&W Scientific, Folsom, CA, USA). The GC conditions were as follows: injector temperature 220 °C; initial oven temperature 60 °C for 1 min, followed by a 120 °C min^−1^ temperature ramp to 180 °C, then a ramp of 50 °C min^−1^ to 200 °C followed by 10.5 min at 200 °C, followed by a ramp of 120 °C min^−1^ to 220 °C. Helium was used as a carrier gas at a flow rate of 1.5 mL/min. FAMEs were detected by a flame ionization detector (FID) set to 220 °C, identified by comparison of their retention times to reference compounds and quantified using previously established calibration curves for each individual FAME (Von Elert, 2002).

### Data analysis

**Figure 1:**
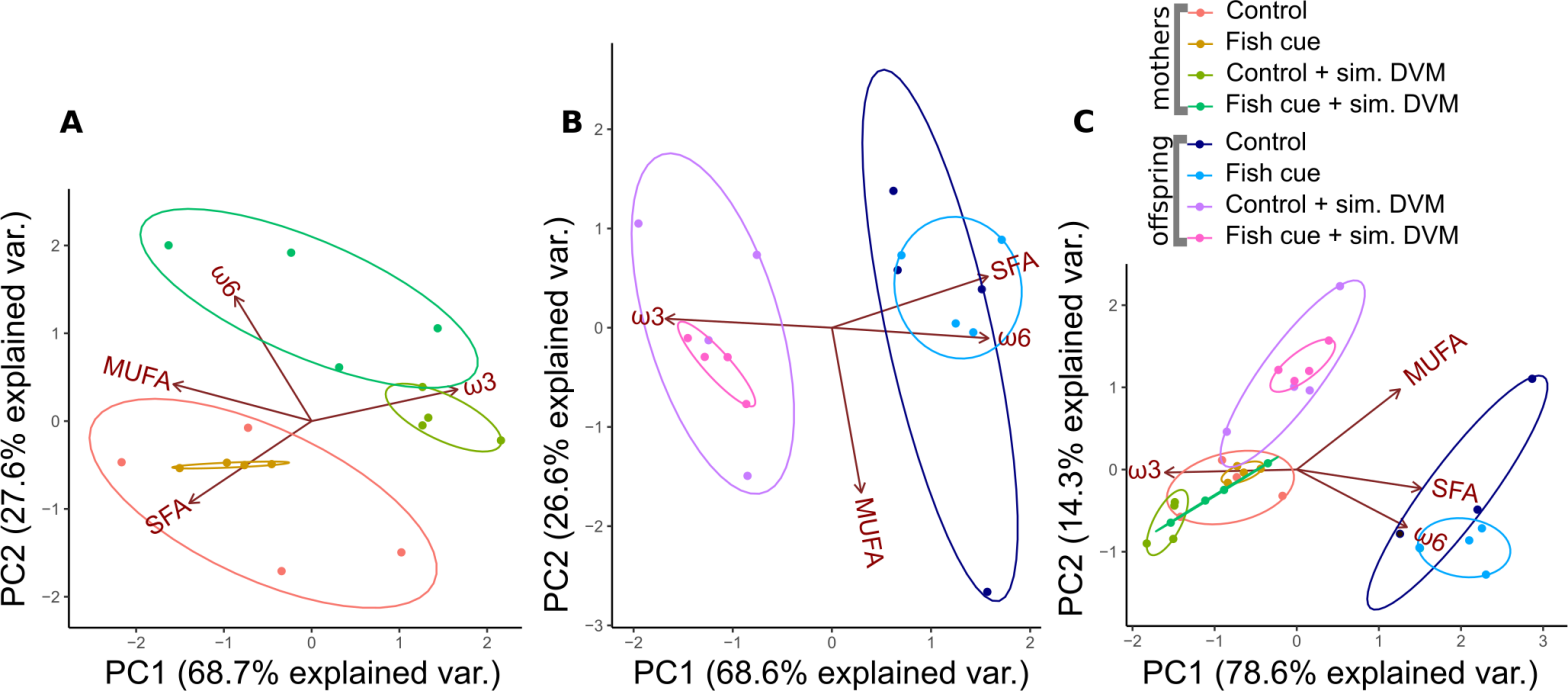
Principal component analysis of relative fatty acid compositions in *Daphnia magna* in controls, treatments with chemical fish cues, controls with simulated DVM and chemical fish cues with simulated DVM. The relative amounts of fatty acid groups (% based on molar concentrations) in (A) *Daphnia magna*, (B) offspring from the 1st clutch and (C) mothers and offspring combined are displayed as ellipses representing 95% confidence intervals, *n* = 4. The percentage of variance explained by principal components 1 and 2 (PC1, PC2) is indicated on the *x*- and *y*-axis.

Data analysis was conducted using the software R (version 3.3.3). Concerning life history parameters and fatty acid concentrations, mean values of all analyzed animals per jar were calculated for further analysis. The mass concentrations of fatty acids were translated into molar concentrations and related to the total fatty acid abundance. To investigate changes of the fatty acid composition of *D. magna*, we grouped the quantified fatty acids by their degree and position of unsaturation, resulting in concentrations for saturated (SFAs), monounsaturated (MUFAs), ω6- and ω3-polyunsaturated fatty acids (PUFAs). We performed principal component analyses (PCA) on the relative distribution of these fatty acid groups in experimental animals (Figs. 1A and 1C) and their offspring (Figs. 1B and 1C) using the package ggbiplot (version 0.55) to create ordination plots. Additionally, permutational MANOVAs (permanovas) and pairwise permanovas were conducted to reveal significant differences considering the factors “simulated DVM”, “fish cue” and in one case the third factor describing the “generation” that *D. magna* originated from. Permanovas were based on Euclidian distance matrices and conducted 1000 permutations. RVAideMemoire package (version 0.9-73) was used for the calculation of permanovas. Prior to the calculation of permanovas homogeneity of multivariate group dispersion was confirmed by ANOVA of the calculated betadispersions using the package vegan (version 2.56).

The resulting data sets were tested for homoscedasticity by Levene’s test using the package car (version 2.1-6) before calculation of a two-way ANOVA testing for effects of the factors “simulated DVM” and “fish cue”. In case of significant effects of the factors or their interaction, Tukey’s HSD test was conducted to reveal statistically different treatments using the package agricolae (version 1.2-8). Results of PCAs, regarding fatty acid groups, were plotted with aid of the package ggbiplot (version 0.55). The allocation of fatty acids from adult animals to their first clutch of offpring was calculated as the amount (mol) of the respective fatty acid in the offspring of one clutch normalized to the sum of the amounts found in the mother and neonates (mol), expressed in percents.

## Results

Simulated diel vertical migration (DVM), in respect of light and temperature alterations or the presence of a predatory chemical cue, impacted the fatty acid composition of *D. magna* on the level of fatty acid groups.

For all three PCAs, the principal components 1 and 2 (PC1, PC2) accounted for more than 93% of the variance in relative fatty acid compositions. In adult females that were exposed/not exposed to simulated DVM, the fatty acid compositions clustered in different groups, which were separated by PC2, accounting for only 27.6% of the variance (Fig. 1A). Animals exposed to simulated DVM were shifted towards a more ω3- and ω6-PUFA dominated fatty acid pattern at the expense of SFAs (Fig. 1A), which were more prominent in animals not exposed to the simulation. *D. magna* that were incubated in presence of the fish cue and underwent simulated DVM tended towards a more ω6-PUFA-rich fatty acid pattern than those which did not perceive a chemical cue (Fig. 1A). The effect of simulated DVM on the maternal fatty acid pattern was confirmed by a permutational MANOVA (permanova; *R*^2^ = 0.77, *p* < 0.001; Table S1A).

In neonates of the first clutch a similar effect of simulated DVM on the fatty acid pattern was found (Fig. 1B). The fatty acid composition of neonates was shifted to a more ω3-PUFA dominated one at the expense of saturated fatty acids and ω6-PUFAs, when their mothers had experienced simulated DVM (Fig. 1B). The relative fatty acid patterns of neonates whose mothers had either been exposed to simulated DVM or not, were mainly separated by PC1, which explained 68.6% of variance. A permanova confirmed the significant effect of simulated DVM on the fatty acid pattern (*R*^2^ = 0.57, *p* < 0.001; Table S1A). In the PCA plot, taking into account the fatty acid patterns of adult *D. magna* and their offspring (Fig. 1C), *D. magna* of all treatments clustered together, except for those neonates, whose ancestors did not experience simulated DVM and who clustered in a second group. This group was shifted towards a more SFA and ω6-PUFA dominated fatty acid pattern. Simulated DVM (*R*^2^ = 0.24, *p* < 0.001; Table S1A), the generation *D. magna* originated from (0.60, *R*^2^ < 0.001; Table S1A), as well as the interaction of both factors (*R*^2^ = 0.05, *p* < 0.01; Table S1A) significantly impacted their fatty acid patterns (permanova). The effect of the generation was the strongest by explaining 60% of the variance.

**Figure 2:**
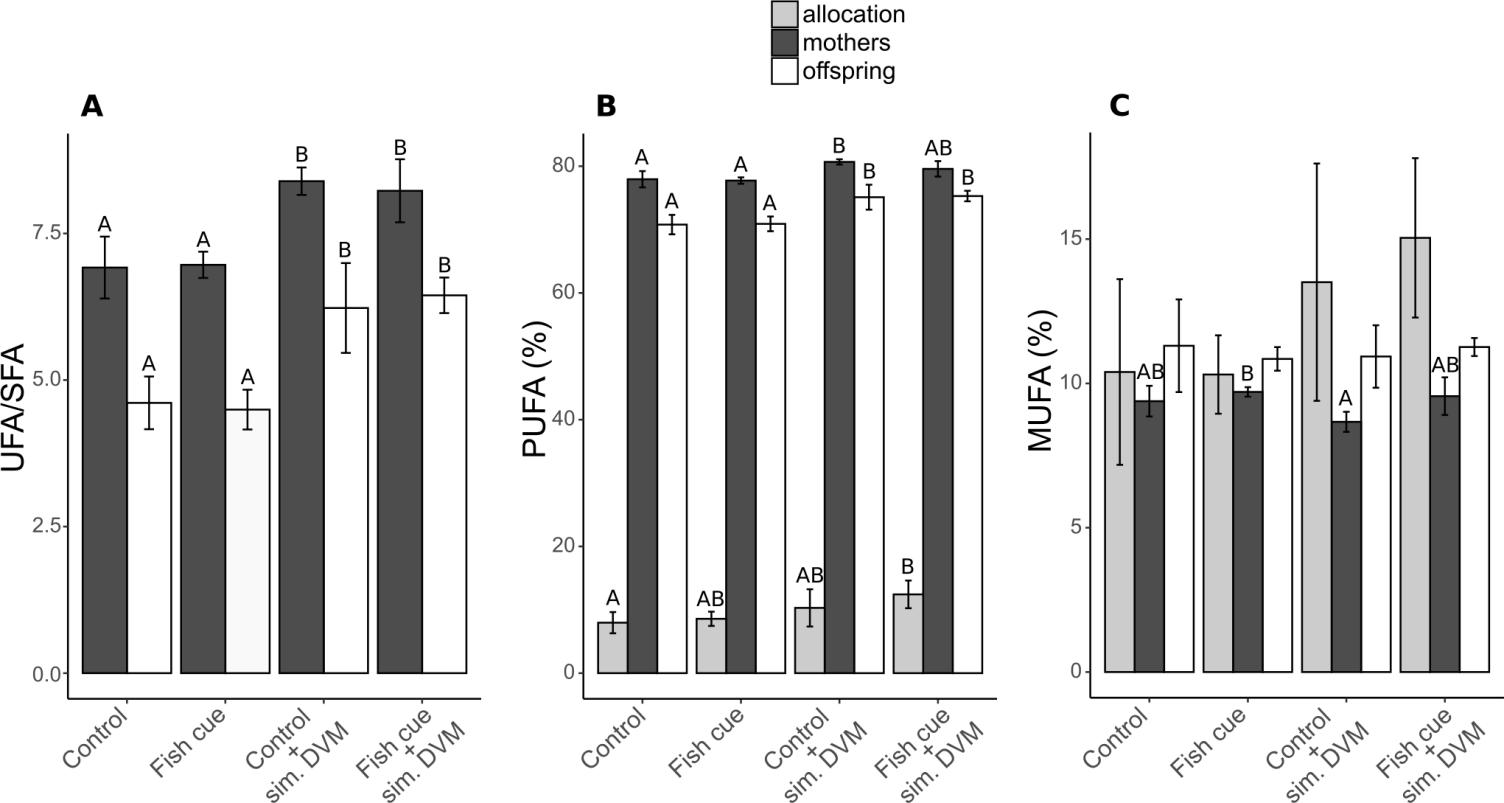
Ratio of unsaturated to saturated fatty acids (UFA/SFA) and the relative compositions and allocations of total polyunsaturated (PUFAs) and monounsaturated fatty acids (MUFAs) in *Daphnia magna*. (A) The ratio of the content of unsaturated fatty acids and saturated fatty acids (UFA/SFA) and (B) the relative PUFA and (C) MUFA concentrations (based on molar concentrations) in adult *D. magna* (darkgrey bars), neonates from their first clutch (white bars), as well as their allocation (grey bars) are depicted. Allocation from mothers to the offspring was calculated as the amount [mol] of the respective fatty acid in all neonates of a clutche normalized to the total amount found in neonates and the maternal animal [mol] and expressed in percent. Depicted are means ± SD after a full factorial life history experiment investigating the factors “fish cue” and “simulated DVM”. Different letters indicate statistically different groups within allocation, mothers, or offspring after two-way ANOVA and Tukey’s HSD test, *n* = 4.

In both, maternal animals and offspring, the UFA/SFA ratio increased due to simulated DVM, being generally lower in the offspring (6.9 vs. 8.4; Fig. 2A, Table S2). The portion of unsaturated fatty acids in experimental animals was built up by PUFAs, accounting for 71–81% of total fatty acids and MUFAs, accounting for 9–11%. The relative PUFA concentration in mothers and their offspring increased with simulated DVM, reflecting the UFA/SFA ratio, with the exception that in adult animals exposed to the fish cue and simulated DVM, the relative PUFA concentration could not be distinguished from that detected in animals that were not undergoing simulated DVM (Fig. 2B, Table S2). PUFA allocation was increased by the simulation of DVM, resulting in a significantly higher PUFA allocation of *D. magna* exposed the fish cue and simulated DVM in comparison to control animals. A pattern was neither detectable in relative MUFA concentrations in neonate *D. magna*, nor in MUFA allocation from maternal animals to their offspring. In maternal animals, the relative MUFA concentration was influenced by the fish cue, resulting in its decrease in animals exposed to simulated DVM without perception of chemical stimuli in comparison to those reared without DVM simulation but in presence of the fish cue (Fig. 2C).

**Figure 3:**
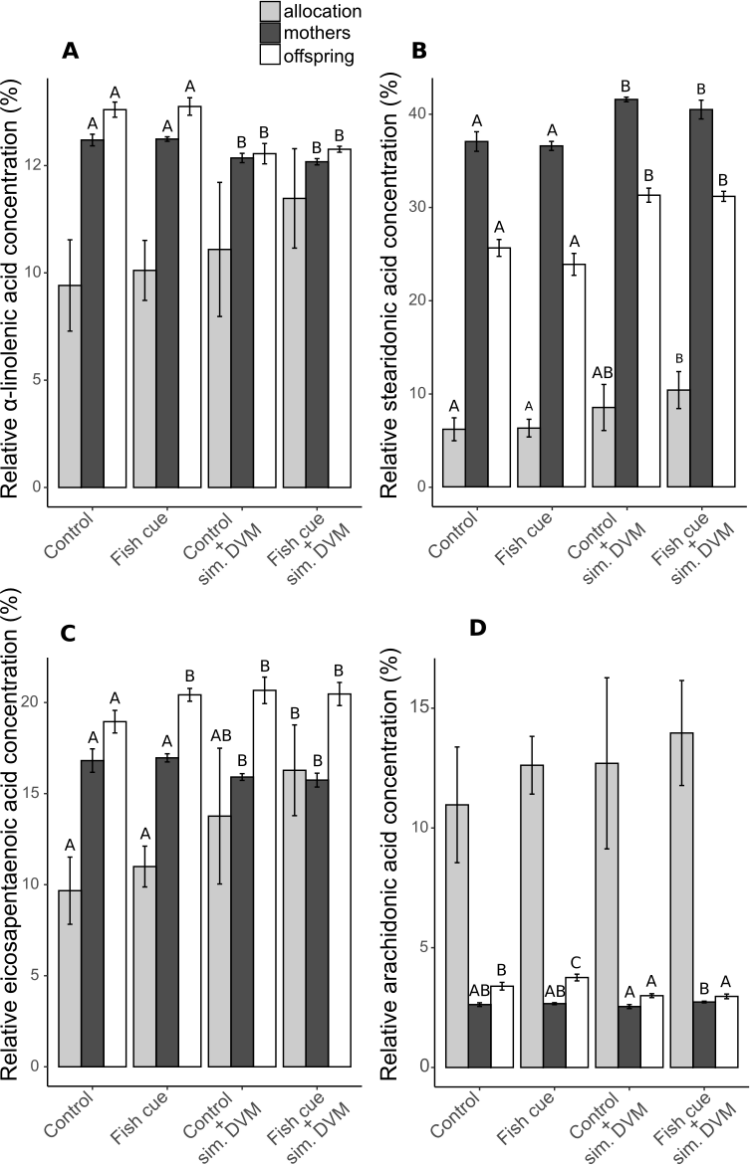
Relative concentrations of ω3- and ω6-PUFAs and their allocations in *Daphnia magna*. The relative concentrations related to the total molar tissue fatty acid content of (A) *α*-linolenic acid (ALA), (B) stearidonic acid (SDA), (C) eicosapentaenoic acid (EPA) and (D) arachidonic acid (ARA) in adult *D-magna* (black bars), neonates from their first clutch (white bars), as well as their allocation from mothers to the offspring (grey bars), expressed as the amount [mol] of the respective fatty acid in all neonates of a clutch normalized to the total amount found in neonates and the maternal animal [mol] and expressed in percent, are displayed. Depicted are mean values ± SD after a full factorial life history experiment investigating the factors “fish cue” and “simulated DVM”. Different letters indicate statistically different treatments among mothers, offspring or allocation after two-way ANOVA and Tukey’s HSD test, *n* = 4.

The described differences in relative PUFA concentrations were partly explained by changes in the relative concentrations of the ω3-PUFAs *α*-linolenic acid (ALA), stearidonic acid (SDA), eicospentaenoic acid (EPA) and of the ω6-PUFA arachidonic acid (ARA) (Fig. 3 and Table S3).

In adult individuals and in their offspring the relative concentration of ALA decreased in response to simulated DVM, while that of SDA increased (Figs. 3A and 3B, Table S3). The relative concentration of EPA was contrarily influenced by simulated DVM in mothers and neonates (Fig. 3C, Table S3). While simulated DVM decreased the relative EPA concentration in mothers, it increased the EPA concentration in their offspring. Additionally, the interaction of the fish cue with simulated DVM impacted the relative EPA concentration in the offspring by increasing the EPA concentration when no DVM was simulated but fish cue perceivable (Fig. 3C and Table S3). SDA and EPA allocation significantly increased with simulated DVM in presence of the fish cue (Figs. 3B & 3C and Table S3). The concentration of the ω6-PUFA ARA in offspring was affected by simulated DVM and the fish cue, as well as by their interaction, yielding elevated ARA concentrations under constant temperatures and an additional elevation due to the fish cue. The fish cue only led to elevated ARA concentrations, when no DVM was simulated (Fig. 3D, Table S3) In mothers ARA concentration was not affected by the mere simulation of DVM, resulting only in elevated ARA concentrations of animals undergoing simulated DVM in presence of the fish cue compared to those not perceiving the cue.

## Discussion

In presence of a chemical cue (i.e., a kairomone) derived from fish, diel vertical migration of *Daphnia* sp. (DVM) is induced as a predator-avoidance behavior (Ringelberg, 1991; Hahn et al., 2019). DVM is accompanied by daily alterations of the animals’ ambient temperatures and decreased mean ambient temperatures (Stich & Lampert, 1981; Dawidowicz & Loose, 1992).

Growth and reproduction of *Daphnia* sp. may be limited by a low content of the essential ω3-PUFA EPA in natural phytoplankton (Müller-Navarra et al., 2000; Wacker & Von Elert, 2001). This EPA-limitation increases at decreasing temperatures (Sperfeld & Wacker, 2012), and additionally EPA limitation suppresses DVM (Brzezinski & Von Elert, 2015). These phenomena were explained by an increased PUFA requirement of animals at lower temperatures to maintain the fluidity of cell membranes, also known as homeoviscous adaptation (Sinensky, 1973; Sperfeld & Wacker, 2012; Brzezinski & Von Elert, 2015). Long-term exposure of *Daphnia pulex* to cold temperatures indeed resulted in elevated relative concentrations of EPA in the animals (Schlechtriem, Arts & Zellmer, 2006). The incorporation of higher concentrations of PUFAs into membranes of poikilothermic animals at low temperatures presumably is adaptive (Hazel & Prosser, 1974), since PUFAs enhance the fluidity of biomembranes. Accordingly, a decreased SFA concentration (increased UFA/SFA ratio) was claimed to be the most common adaptation of poikilotherms to decreasing temperatures (Hazel & Williams, 1990). We hypothesized that the simulation of DVM in terms of ambient light and temperature would change the fatty acid composition in *D. magna* and their offspring in a way that would allow to maintain membrane fluidity at decreased average temperatures. We expected increased relative PUFA concentrations under simulated DVM to keep membrane fluidity constant at decreased average temperatures. An increased EPA demand in this context might explain, why EPA availability constrains DVM (Brzezinski & Von Elert, 2015). As hypothesized, the observed increase of the UFA/SFA ratio in mothers and their offspring, undergoing simulated DVM, implies increased membrane fluidity under lower average, but fluctuating temperatures (Hazel & Williams, 1990). This suggests homeoviscous adaptation. The increase of the UFA/SFA ratio does not fully corroborate an earlier study, in which this increased ratio was not detected under fluctuating temperatures, although total PUFA concentrations increased (Isanta Navarro et al., 2019). This discrepancy appeared most probably due to different light, food and temperature conditions. Additionally, in the here presented study, maternal animals were reared for 14 to 18 days in the simulated DVM treatment before fatty acids were analyzed, in order to allow for physiological acclimation. The acclimation time until fatty acid sampling was longer than in the study of Isanta Navarro et al. (2019) (six days) and may have been necessary to allow for manifestation of temperature effects on the UFA/SFA ratio.

In our study the increase in UFA/SFA in response to simulated DVM can be attributed to changes in relative PUFA concentrations (offspring) and changes in the sum of PUFA and MUFA concentrations (mothers). Zeis et al. (2019) showed that there was no difference in relative PUFA, MUFA and SFA concentrations, when animals were acclimated to constant temperatures of 10 or 20 °C. The fact that *D. magna* acclimated to 10 and 20 °C did not show different PUFA concentrations (Zeis et al., 2019), whereas here changed PUFA concentrations have been observed in animals exposed to average temperatures of a very similar range (12.5 and 21 °C), strongly suggests that the simulated DVM may impact fatty acid composition in *D. magna* in other ways than by a decreased mean temperature only. The fluctuations in ambient temperature and the darkness are potential factors which could have caused the increased PUFA concentrations. Isanta Navarro et al. (2019) showed that in a non-EPA supplemented treatment, PUFA concentrations increased due to temperature fluctuations, which is well in accordance with the here reported general PUFA increase under simulated DVM. This finding supports the reasoning that simulated DVM mainly affects *D. magna* fatty acid composition by fluctuating temperatures rather than by the absence of light. It was shown that an increasing ratio of unsaturated fatty acids to saturated fatty acids (UFA/SFA) correlates well with membrane fluidity, assessed by fluorescence polarization (Cossins & Prosser, 1978; Hazel & Williams, 1990). A weaker correlation was observed for membrane fluidity and the so-called unsaturation index (UI) (Cossins & Prosser, 1978). The UI calculated by (Cossins & Prosser, 1978) provides the relative number of double bonds per fatty acid, relating the number of double bonds to total fatty acids. Saturated fatty acids (SFAs) are included in their calculations. They therewith acknowledge that (i) SFAs affect membrane fluidity negatively and (ii) that fatty acid composition and not its content per biomass is sufficient to explain membrane fluidity. In line with this, we here have calculated an UI that weighs unsaturated fatty acids according to their number of double bonds and relates them to total fatty acids, thus accounting for the share of saturated fatty acids (Fig. S2, Table S4). We report that this UI and the UFA/SFA ratio increased in *D. magna* exposed to simulated DVM. According to (Cossins & Prosser, 1978; Hazel & Williams, 1990) this suggests increased membrane fluidity in *D. magna* exposed to simulated DVM. However, we have not assessed membrane fluidity. Only in (Martin-Creuzburg et al., 2019) membrane fluidity and fatty acids have been determined in *D. magna*. In that study an UI was calculated that was, contrary to the one calculated here, based on fatty acid desaturation related to animal biomass. That index does consequently not consider fatty acid composition and not account for the concentration of SFAs, which was claimed to be the most common predictor of membrane fluidity (Hazel & Williams, 1990). Still, the calculated UI correlated with membrane fluidity (Martin-Creuzburg et al., 2019). In conclusion, the here observed significant changes in UI and UFA/SFA ratio under simulated DVM (i.e., decreased average temperatures) are in agreement with homeoviscous adaptation of *D. magna*.

However, in contrast to our hypothesis, simulated DVM decreased the relative EPA content in maternal animals, while it increased the content in the offspring. In a study of Isanta Navarro et al. (2019) investigating the effect of temperature fluctuations and dietary EPA supply on performance and fatty acid composition of *D. magna*, food supplementation with EPA liposomes led to increased tissue concentrations of EPA in maternal animals exposed to temperature fluctuations. Although this result seems to contradict our finding of decreased relative EPA concentrations of maternal animals undergoing simulated DVM, comparison of both experimental set-ups may explain the different findings. First of all, we here report relative molar EPA concentrations, which is not comparable to the tissue concentrations described by Isanta Navarro et al. (2019). Secondly, the authors extracted fatty acids of animals before releasing the first clutch, so that a potential redistribution of EPA into eggs and mothers is not considered. Differences with respect to food sources, light regimes, temperatures and EPA supplementation through liposomes might further explain the observed differences in EPA concentrations. As a low EPA supply constrains DVM (Brzezinski & Von Elert, 2015), the increase of EPA in offspring at the expense of maternal EPA, may be interpreted as a resource allocation from the mothers to their offspring that is adaptive for the offspring. Sperfeld & Wacker (2012) showed that at low temperatures EPA concentrations increased in adult *D. magna* but not in their offspring. Apparently, this pattern cannot be transferred to *D. magna* exposed to fluctuating and thereby lower average temperatures and darkness, as they are experienced during DVM.

Our results for the first time suggest that homeoviscous adaptation might also occur in offspring of animals exposed to darkness and temperature fluctuations as observed during DVM. This adaptation can be explained by decreased mean temperatures experienced by the migrating animals. It is reasonable to assume that this trans-generational change in fatty acid composition is adaptive, as DVM in lakes usually occurs over several months during the stratification period (Stich, 1989; Ringelberg, 1991) so that the offspring of DVM-performing *D. magna* will as well be deploying DVM.

Furthermore, we show that the DVM inducing fish cue as well impacts the fatty acid pattern of *D. magna*. The fish cue was found to increase the relative concentrations of the ω6-PUFA ARA in *D. magna.* However, we can only speculate about its adaptive value. In invertebrates ARA is known to be a precursor for several signaling molecules (prostaglandins, leukotrienes and thromboxanes) (Schlotz, Sorensen & Martin-Creuzburg, 2012). In some crustaceans ARA was identified as the precursor of several prostaglandins involved in reproductive processes (Tahara & Yano, 2003; Rowley et al., 2005) as for instance the induction of ovulation (Spaziani, Hinsch & Edwards, 1993). In *D. magna* feeding on sterol and PUFA lacking *Synechococcus elongatus*, supplementation of ARA at increasing sterol concentrations significantly increased the population growth rate (Martin-Creuzburg, Wacker & Basena, 2010). The population growth rate was as well increased by ARA supplementation, when *D. magna* fed on *Acutodesmus obliquus* (lacking C_20_ PUFAs) on a temperature gradient, where the supplementation had a higher effect at lower temperatures (Martin-Creuzburg et al., 2012). In six cladoceran species the bioconversion and accumulation of several PUFAs, including ARA, was claimed to be on average higher at 14 than at 20 °C (Masclaux et al., 2012). These findings might suggest a role of ARA in adaptation to decreased temperatures. Interestingly, the fish cue increased the relative EPA concentration in offspring when no DVM was simulated. One might speculate that this increase in EPA concentration constitutes a pre-adaptation for DVM after hatching, since it was shown that low dietary EPA limits DVM in *D. magna* (Brzezinski & Von Elert, 2015) and EPA supplementation increases the fitness (somatic growth and population growth rate) of individuals exposed to temperature fluctuations (Isanta Navarro et al., 2019). The observation that the chemical fish cue triggered increased relative EPA concentrations only in offspring not exposed to simulated DVM (i.e., not exposed to darkness and alternating temperatures), suggests that the fish cue serves as a trigger for increased EPA-concentrations only when *D. magna* perceives light. The finding that the fish cue affects EPA concentration only in the presence of light nicely corresponds to the kairomone-induced onset of DVM and of life history changes in *D. magna*, each of which requires both the kairomone and light (Loose, 1993; Effertz & Von Elert, 2014; Effertz & Von Elert, 2017). Our simulated DVM treatment comprised entire darkness. However, during DVM *Daphnia* sp. will distribute vertically according to ideal free distribution with costs, i.e., not all individuals will stay at the same depth (Larsson & Lampert, 2012), with a few of them temporarily being exposed to slightly higher light levels. Hence, in nature effects of kairomone on the relative EPA-concentration of offspring may even occur in DVM-performing animals.

The conversion of ALA to EPA in *Daphnia* sp. has been demonstrated repeatedly (Weers, Siewersen & Gulati, 1997; Von Elert, 2002; Taipale, Kainz & Brett, 2011) and is probably achieved via the synthesis of the intermediate product SDA (Von Elert, 2002). Our results strongly indicate elevated conversion rates of ALA to SDA under simulated DVM, since simulated DVM decreased the relative concentration of ALA and increased that of SDA. Astonishingly, the suggested increased conversion did not result in an increase of its end product EPA in adult *D. magna*. Still, this putatively increased conversion of ω3-PUFAs to EPA might be overseen due to increased EPA allocations from mothers to their offspring. Furthermore, it is disputable if differential fatty acid compositions in the offspring in comparison to their mothers may be solely attributed to differential resource allocation.

In our experimental setup, offspring hatched into the maternal feeding environment. Thus, juveniles were allowed to feed on *Cryptomonas* sp. for 8 h on average, before they were sampled for fatty acid analysis. To avoid potential effects of this feeding on fatty acid composition in offspring, eggs could have been dissected from mothers (Sperfeld & Wacker, 2012) or mothers could have been transferred to food-free media prior to hatching. Still, the finding that the same treatment (simulated DVM) had opposite effects on EPA concentrations in neonates and mothers strongly suggests that EPA concentration increases in offspring on the expense of EPA concentration in mothers and thus supports our interpretation that the fatty acids measured in offspring mainly resulted from maternal resource allocation.

Although we report about strong effects of simulated DVM on fatty acid patterns in *D. magna*, it should still be drawn attention to the fact that we here specified simulated DVM as the simulation of light and temperature conditions only. It remains to be investigated how other parameters, which as well fluctuate during DVM (e.g., food and oxygen levels) would affect *Daphnia* fatty acid composition. Furthermore, the mere extraction of membrane lipids would allow for a clearer statement about homeoviscous adaptation than extraction of whole body fatty acids, since homeoviscous adaptation occurs on the level of biomembranes.

## Conclusions

We found that both the simulation of DVM in terms of light and temperature regime and the exposure to the fish cue, resulted in changes in the fatty acid composition of *D. magna* that are known to be adaptive at decreased mean temperatures and thus assumed to be adaptive, when DVM is performed (increased UFA/SFA ratio, ARA concentration). Simulated DVM and the fish cue in absence of simulated DVM induced trans-generational effects, resulting in an increased concentration of the ω3-PUFA EPA in offspring. DVM performing animals that perceive the fish cue, exhibit a change in fatty acid allocation. Since EPA supplementation at fluctuating temperatures is known to increase the somatic and the population growth rate of *D. magna*, this change can be assumed to be adaptive for animals of the next generation if they keep on performing DVM after hatching.

##  Supplemental Information

10.7717/peerj.8809/supp-1 Figure S1 Daily temperature profile (°C) in the waterbath simulating diel vertical migration (DVM) Depicted are the mean values of 4 individual measurements from each corner of the water bath (*n* = 4). Standard deviations did not exeed 0.3 °C.

10.7717/peerj.8809/supp-2 Figure S2 Unsaturation index (UI) of fatty acids of *D. magna* and their offspring exposed to the factors “simulated DVM” and “fish cue” UI is calculated as the relative sum of single fatty acids (mol of total fatty acids) multiplied by their degree of unsaturation (no. of double bonds). Depicted are means ± SD after a full-factorial life history experiment investigating the factors “fish cue” and “simulated DVM”. Different letters indicate statistically differing groups within mothers, or offspring after two-way ANOVA and Tukey’s HSD test, *N* = 4.

10.7717/peerj.8809/supp-3 Figure S3 Life history parameters of *Daphnia magna* in response to the factors “fish cue” and “simulated DVM” The life history parameters A growth rate [day^−1^], B age at first reproduction [days], C individual weight, D size at first reproduction, E clutch size and F the clutch weight as percentage of the maternal weight od *Daphnia magna* after a full-factorial bioassay investigating the factors “*fish cue*” and “*simulated DVM*” are depicted as means ± SD. “*Control*” treatment (white bars), “*fish cue*” treatment (black bars), the “*simulated DVM*” in absence (light-grey bars) and in presence of the fish cue (dark-grey bars) are grouped. Different letters indicate statistically differing groups after two-way ANOVA and Tukey’s HSD test.

10.7717/peerj.8809/supp-4 Table S1A Results of permutational MANOVAs on the effect of *simulated DVM, fish cue* and *generation* on the fatty acid group composition in *Daphnia magna* Significant effects (*p* < 0.05) are highlighted in bold.

10.7717/peerj.8809/supp-5 Table S1B Results of pairwise permutational MANOVAs on the effect of *simulated DVM, fish cue* and *generation* on the fatty acid group composition in *Daphnia magna* Significant effects (*p* < 0.05) are highlighted in bold.

10.7717/peerj.8809/supp-6 Table S2 Results of Two-Way ANOVAs on the effect of “simulated DVM” and “fish cue” on the ratio of unsaturated and saturated fatty acids (UFA/SFA) and the relative concentrations of total polyunsaturated fatty acids and monounsaturated fatty acids (%) based on molar concentrations in Daphnia magna, their offspring and the percentual allocation calculated as the percentage of the respective amount [ng] per neonate of the total amount found in neonates and maternal animals [ng]. Significant effects (*p* < 0.05) are highlighted in bold, *N* = 4.

10.7717/peerj.8809/supp-7 Table S3 Results of Two-Way ANOVAs on the effect of the factors ‘simulated DVM’ and ‘fish cue’ on the relative molar concentration of the ω3- and ω6-PUFAs *α*-linolenic acid (ALA), stearidonic acid (SDA), eicosa pentaenoic acid (EPA) and arachidonic acid Significant effects are highlighted in bold, *N* = 4.

10.7717/peerj.8809/supp-8 Table S4 Results of Two-Way ANOVAs on the effect of the factors ‘simulated DVM’ and ‘fish cue’ on the relative unsaturation index (UI) in Daphnia magna and their offspring. UI was calculated as the sum of the relative molar concentration of each fatty acid multiplied by its respective number of double bonds. Significant effects are highlighted in bold, *N* = 4.

10.7717/peerj.8809/supp-9 Data S1 Fatty acid concentrations and animal weight 1

10.7717/peerj.8809/supp-10 Data S2 Fatty acid concentrations and animal weight 2

## Additional Information and Declarations

### Data Availability

The following information was supplied regarding data availability: The raw measurements regarding fatty acid concentrations and animal weight are available in the Supplemental Files.
