# Supplementary figures and images for: The impact of diel vertical migration on fatty acid patterns and allocation in Daphnia magna
Source: PeerJ. 2020 Apr 17;8:e8809. doi: 10.7717/peerj.8809 (PMC7169964; doi:10.7717/peerj.8809)

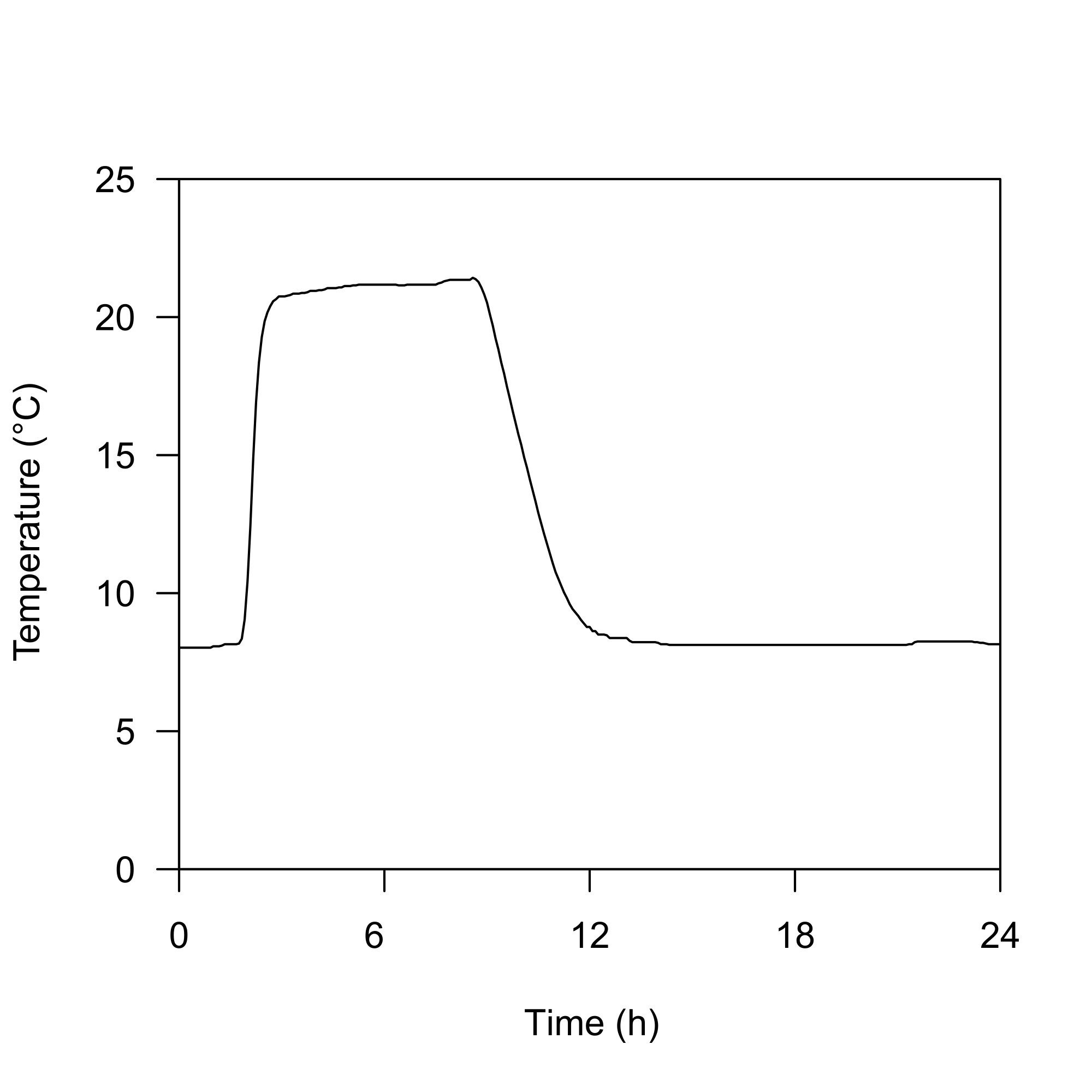

Supplement: Figure S1 — Depicted are the mean values of 4 individual measurements from each corner of the water bath (n = 4). Standard deviations did not exeed 0.3 °C. [file peerj-08-8809-s001.png]

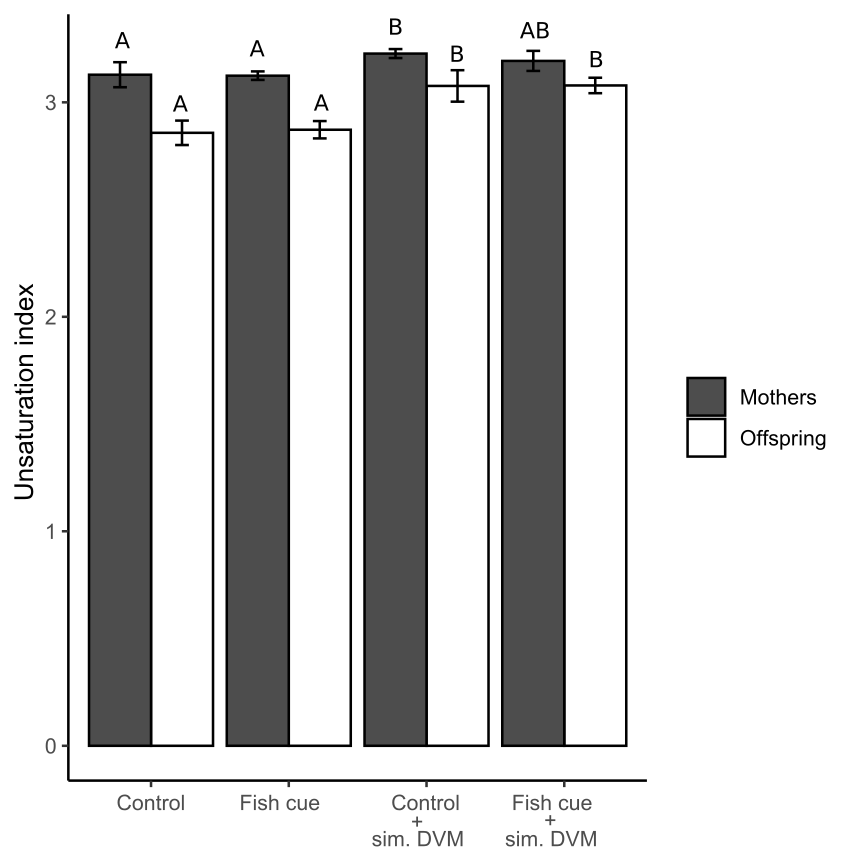

Supplement: Figure S2 — UI is calculated as the relative sum of single fatty acids (mol of total fatty acids) multiplied by their degree of unsaturation (no. of double bonds). Depicted are means ± SD after a full-factorial life history experiment investigating the factors “fish cue” and “simulated DVM”. Different letters indicate statistically differing groups within mothers, or offspring after two-way ANOVA and Tukey’s HSD test, N = 4. [file peerj-08-8809-s002.png]

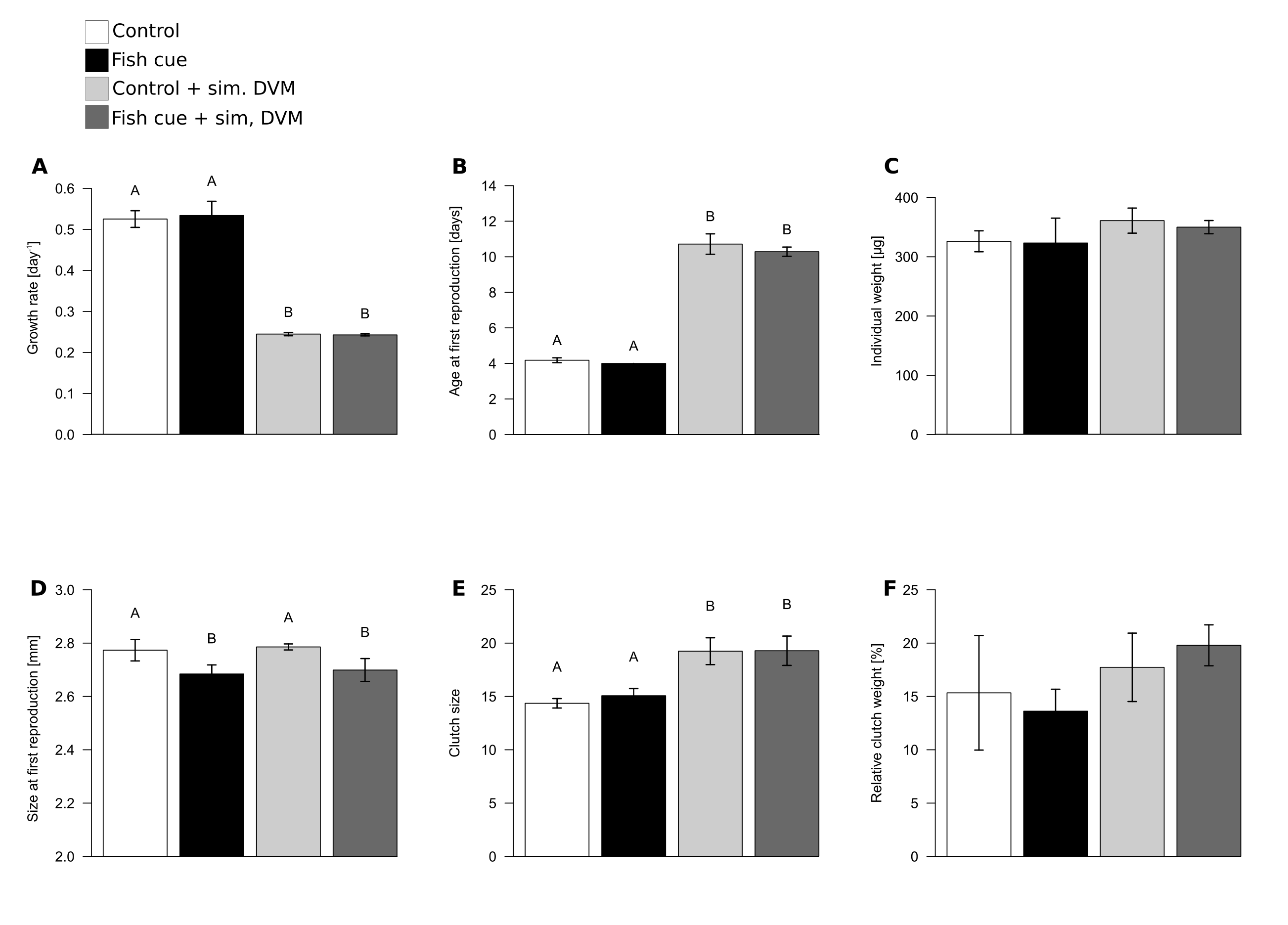

Supplement: Figure S3 — The life history parameters A growth rate [day−1], B age at first reproduction [days], C individual weight, D size at first reproduction, E clutch size and F the clutch weight as percentage of the maternal weight od Daphnia magna after a full-factorial bioassay investigating the factors “fish cue” and “simulated DVM” are depicted as means ± SD. “Control” treatment (white bars), “fish cue” treatment (black bars), the “simulated DVM” in absence (light-grey bars) and in presence of the fish cue (dark-grey bars) are grouped. Different letters indicate statistically differing groups after two-way ANOVA and Tukey’s HSD test. [file peerj-08-8809-s003.png]
