# Supplementary material for: The impact of diel vertical migration on fatty acid patterns and allocation in Daphnia magna
Source: PeerJ. 2020 Apr 17;8:e8809. doi: 10.7717/peerj.8809 (PMC7169964; doi:10.7717/peerj.8809)
Supplement: Table S1A — Significant effects (p < 0.05) are highlighted in bold. [file peerj-08-8809-s004.docx]

**SI Table 1A:** Results of permutational MANOVAs on the effect of *simulated DVM,*  *fish cue* and *generetion* on the fatty acid group composition in *Daphnia magna*. Significant effects (p<0.05) are highlighted in bold.

| **mothers** | **Df** | **SS** | **MS** | **F** | **R2** | **p-value** |  |
| --- | --- | --- | --- | --- | --- | --- | --- |
| **simulated DVM** | 1 | 33.931 | 33.931 | 20.3608 | 0.56746 | **0.000999** | *** |
| fish cue | 1 | 4.216 | 4.216 | 2.5299 | 0.07051 | 0.11988 |  |
| simulated DVM x fish cue | 1 | 1.649 | 1.649 | 0.9897 | 0.02758 | 0.352647 |  |
| residuals | 12 | 19.998 | 1.667 |  | 0.33444 |  |  |
| total | 15 | 59.795 |  |  | 1 |  |  |
| **Offspring** | **Df** | **SS** | **MS** | **F** | **R2** | **p-value** |  |
| **simulated DVM** | 1 | 202.32 | 202.32 | 42.276 | 0.77432 | **0.000999** | *** |
| fish cue | 1 | 0.177 | 0.177 | 0.037 | 0.00068 | 0.955045 |  |
| simulated DVM x fish cue | 1 | 1.361 | 1.361 | 0.284 | 0.00521 | 0.631369 |  |
| residuals | 12 | 57.427 | 4.786 |  | 0.21979 |  |  |
| total | 15 | 261.28 |  |  | 1 |  |  |
| **mothers+offspring** | **Df** | **SS** | **MS** | **F** | **R2** | **p-value** |  |
| **sim. DVM** | 1 | 198.48 | 198.48 | 61.526 | 0.24493 | **0.000999** | *** |
| fish cue | 1 | 2.23 | 2.23 | 0.692 | 0.00275 | 0.436563 |  |
| **generation** | 1 | 489.3 | 489.3 | 151.671 | 0.60379 | **0.000999** | *** |
| sim. DVM x fish cue | 1 | 1.52 | 1.52 | 0.471 | 0.00187 | 0.517483 |  |
| **sim. DVM x generation** | 1 | 37.76 | 37.76 | 11.706 | 0.0466 | **0.002997** | ** |
| fish cue x generation | 1 | 2.16 | 2.16 | 0.67 | 0.00267 | 0.406593 |  |
| sim. DVM x fish cue x generation | 1 | 1.49 | 1.49 | 0.462 | 0.00184 | 0.528472 |  |
| Residuals | 24 | 77.43 | 3.23 |  | 0.09554 |  |  |
| Total | 31 | 810.38 |  |  | 1 |  |  |
