# Supplementary material for: The impact of diel vertical migration on fatty acid patterns and allocation in Daphnia magna
Source: PeerJ. 2020 Apr 17;8:e8809. doi: 10.7717/peerj.8809 (PMC7169964; doi:10.7717/peerj.8809)
Supplement: Table S1B — Significant effects (p < 0.05) are highlighted in bold. [file peerj-08-8809-s005.docx]

**SI Table 1B:** Results of pairwise permutational MANOVAs on the effect of *simulated DVM,*  *fish cue* and *generetion* on the fatty acid group composition in *Daphnia magna*. Significant effects (p<0.05) are highlighted in bold.

| **mothers** | control | fish cue | control + sim. DVM |  |  |  |  |
| --- | --- | --- | --- | --- | --- | --- | --- |
| fish cue | 0.808 | - | - |  |  |  |  |
| control + sim. DVM | 0.092 | 0.092 | - |  |  |  |  |
| fish cue + sim. DVM | 0.092 | 0.102 | 0.15 |  |  |  |  |
| **offspring** | control | fish cue | control + sim. DVM |  |  |  |  |
| fish cue | 0.918 | - | - |  |  |  |  |
| control + sim. DVM | 0.055 | 0.055 | - |  |  |  |  |
| fish cue + sim. DVM | 0.055 | 0.055 | 0.918 |  |  |  |  |
| **mothers + offspring** | control | fish cue | sim. DVM + control | sim. DVM + fish cue | control + offspring | fish cue + offspring | sim. DVM + control + offspring |
| fish cue | 0.89 | - | - | - | - | - | - |
| sim. DVM+control | 0.051 | 0.051 | - | - | - | - | - |
| DVMF | 0.073 | 0.069 | 0.112 | - | - | - | - |
| control + offspring | 0.051 | 0.051 | 0.051 | 0.055 | - | - | - |
| fish cue + offspring | 0.051 | 0.051 | 0.051 | 0.051 | 0.918 | - | - |
| sim. + control+ offspring | 0.069 | 0.101 | 0.051 | 0.051 | 0.051 | 0.051 | - |
| sim. DVM + fish cue + offspring | 0.051 | 0.053 | 0.051 | 0.051 | 0.051 | 0.051 | 0.918 |
