# Supplementary material for: The impact of diel vertical migration on fatty acid patterns and allocation in Daphnia magna
Source: PeerJ. 2020 Apr 17;8:e8809. doi: 10.7717/peerj.8809 (PMC7169964; doi:10.7717/peerj.8809)
Supplement: Table S2 — Results of Two-Way ANOVAs on the effect of “simulated DVM” and “fish cue” on the ratio of unsaturated and saturated fatty acids (UFA/SFA) and the relative concentrations of total polyunsaturated fatty acids and monounsaturated fatty acids (%) based on molar concentrations in Daphnia magna, their offspring and the percentual allocation calculated as the percentage of the respective amount [ng] per neonate of the total amount found in neonates and maternal animals [ng]. Significant effects (p < 0.05) are highlighted in bold, N = 4. [file peerj-08-8809-s006.docx]

SI Table 2: Results of Two-Way ANOVAs on the effect of “simulated DVM” and “ fish cue” on the ratio of unsaturated and saturated fatty acids (UFA/SFA) and the relative concentrations of total polyunsaturated fatty acids and monounsaturated fatty acids (%) based on molar concentrations in *Daphnia magna*, their offspring and the percentual allocation calculated as the percentage of the respective amount [ng] per neonate of the total amount found in neonates and maternal animals [ng] . Significant effects (p<0.05) are highlighted in bold, N=4.

| **UFA/SFA** | **df** | **SS** | **MS** | **F** | **p-value** |  | **mothers** |
| --- | --- | --- | --- | --- | --- | --- | --- |
| simulated DVM | 1 | 7.485 | 7.485 | 4.45E+01 | **2.28E-05** | *** |  |
| fish cue | 1 | 0.014 | 0.014 | 0.082 | 0.779 |  |  |
| simulated DVM x fish cue | 1 | 0.044 | 0.044 | 0.263 | 0.617 |  |  |
| residuals | 12 | 2.017 | 0.168 |  |  |  |  |
| **PUFA** | **df** | **SS** | **MS** | **F** | **p-value** |  |  |
| simulated DVM | 1 2 | 0.997 | 20.997 | 23.499 | **0.0004** | *** |  |
| fish cue | 1 | 1.718 | 1.718 | 1.923 | 0.1907 |  |  |
| simulated DVM x fish cue | 1 | 0.793 | 0.793 | 0.888 | 0.3647 |  |  |
| residuals | 12 1 | 0.722 | 0.894 |  |  |  |  |
| **MUFA** | **df** | **SS** | **MS** | **F** | **p-value** |  |  |
| simulated DVM | 1 0 | 0.7467 | 0.7467 | 3.488 | 0.0864 |  |  |
| fish cue | 1 1 | 0.4635 | 1.4635 | 6.836 | **0.0226** | * |  |
| simulated DVM x fish cue | 1 0 | 0.3234 | 0.3234 | 1.511 | 0.2426 |  |  |
| residuals | 12 2 | 0.5692 | 0.2141 |  |  |  |  |
| **UFA/SFA** | **df** | **SS** | **MS** | **F** | **p-value** |  | **offspring** |
| simulated DVM | 1 | 12.731 | 12.731 | 51.128 | **1.16E-05** | *** |  |
| fish cue | 1 | 0.01 | 0.01 | 0.041 | 0.842 |  |  |
| simulated DVM x fish cue | 1 | 0.11 | 0.11 | 0.44 | 0.52 |  |  |
| residuals | 12 | 2.988 | 0.249 |  |  |  |  |
| **PUFA** | **df** | **SS** | **MS** | **F** | **p-value** |  |  |
| simulated DVM | 1 | 75.95 | 75.95 | 36.899 | **5.55E-05** | *** |  |
| fish cue | 1 | 0.09 | 0.09 | 0.044 | 0.838 |  |  |
| simulated DVM x fish cue | 1 | 0 | 0 | 0.002 | 0.964 |  |  |
| residuals | 12 | 24.7 | 2.06 |  |  |  |  |
| **MUFA** | **df** | **SS** | **MS** | **F** | **p-value** |  |  |
| simulated DVM | 1 | 0.002 | 0.0016 | 0.002 | 0.969 |  |  |
| fish cue | 1 | 0.016 | 0.0162 | 0.016 | 0.901 |  |  |
| simulated DVM x fish cue | 1 | 0.616 | 0.6158 | 0.614 | 0.449 |  |  |
| residuals | 12 | 12.042 | 1.0035 |  |  |  |  |
| **PUFA** | **df** | **SS** | **MS** | **F** | **p-value** |  | **relative allocation** |
| simulated DVM | 1 | 38.32 | 38.32 | 8.785 | **0.0118** | * |  |
| fish cue | 1 | 7.52 | 7.52 | 1.723 | 0.2139 |  |  |
| simulated DVM x fish cue | 1 | 2.29 | 2.29 | 0.525 | 0.4826 |  |  |
| residuals | 12 | 52.35 | 4.36 |  |  |  |  |
| **MUFA** | **df** | **SS** | **MS** | **F** | **p-value** |  |  |
| simulated DVM | 1 | 61.58 | 61.58 | 6.707 | **0.0237** | * |  |
| fish cue | 1 | 2.1 | 2.1 | 0.228 | 0.6413 |  |  |
| simulated DVM x fish cue | 1 | 2.63 | 2.63 | 0.287 | 0.602 |  |  |
| residuals | 12 | 110.17 | 9.18 |  |  |  |  |
