# Supplementary material for: The impact of diel vertical migration on fatty acid patterns and allocation in Daphnia magna
Source: PeerJ. 2020 Apr 17;8:e8809. doi: 10.7717/peerj.8809 (PMC7169964; doi:10.7717/peerj.8809)
Supplement: Table S3 — Significant effects are highlighted in bold, N = 4. [file peerj-08-8809-s007.docx]

SI Table 5: Results of Two-Way ANOVAs on the effect of the factors ‘simulated DVM’ and ‘fish cue’ on the relative molar concentration of the ω3- and ω6-PUFAs α-linolenic acid (ALA), stearidonic acid (SDA), eicosa pentaenoic acid (EPA) and arachidonic acid (ARA) in *Daphnia magna*, their offspring and the percentual allocation expressed as the percentage of the respective amount [ng] per neonate of the amount [ng] of the total amount found in neonates and maternal animals [ng]. Significant effects are highlighted in bold, N=4.

| **ALA** | **df** | **SS** | **MS** | **F** | **p-value** |  | **mothers** |
| --- | --- | --- | --- | --- | --- | --- | --- |
| simulated DVM | 1 | 3.587 | 3.587 | 94.151 | **4.95E-07** | *** |  |
| fish cue | 1 | 0.017 | 0.017 | 0.446 | 0.517 |  |  |
| simulated DVM x fish cue | 1 | 0.049 | 0.049 | 1.276 | 0.281 |  |  |
| residuals | 12 | 0.457 | 0.038 |  |  |  |  |
| **SDA** | **df** | **SS** | **MS** | **F** | **p-value** |  |  |
| simulated DVM | 1 | 70.78 | 70.78 | 117.47 | **1.49E-07** | *** |  |
| fish cue | 1 | 2.42 | 2.42 | 4.022 | 0.068 | . |  |
| simulated DVM x fish cue | 1 | 0.38 | 0.38 | 0.632 | 0.442 |  |  |
| residuals | 12 | 7.23 | 0.6 |  |  |  |  |
| **EPA** | **df** | **SS** | **MS** | **F** | **p-value** |  |  |
| simulated DVM | 1 | 4.505 | 4.505 | 27.991 | **0.000191** | *** |  |
| fish cue | 1 | 0 | 0 | 0.002 | 0.964788 |  |  |
| simulated DVM x fish cue | 1 | 0.102 | 0.102 | 0.633 | 0.441552 |  |  |
| residuals | 12 | 1.932 | 0.161 |  |  |  |  |
| **ARA** | **df** | **SS** | **MS** | **F** | **p-value** |  |  |
| simulated DVM | 1 0 | 0.0002 | 0.0002 | 0.053 | 0.82261 |  |  |
| fish cue | 1 0 | 0.05319 | 0.05319 | 13.703 | **0.00303** | ** |  |
| simulated DVM x fish cue | 1 0 | 0.02282 | 0.02282 | 5.878 | **0.03205** | * |  |
| residuals | 12 0 | 0.04658 | 0.00388 |  |  |  |  |
| **ALA** | **df** | **SS** | **MS** | **F** | **p-value** |  | **offspring** |
| simulated DVM | 1 | 16.357 | 16.357 | 122.396 | **1.19E-07** | *** |  |
| fish cue | 1 | 0.124 | 0.124 | 0.928 | 0.354 |  |  |
| simulated DVM x fish cue | 1 | 0.003 | 0.003 | 0.026 | 0.875 |  |  |
| residuals | 12 | 1.604 | 0.134 |  |  |  |  |
| **SDA** | **df** | **SS** | **MS** | **F** | **p-value** |  |  |
| simulated DVM | 1 | 167.62 | 167.62 | 218.642 | **4.58E-09** | *** |  |
| fish cue | 1 | 3.56 | 3.56 | 4.644 | 0.0522 | . |  |
| simulated DVM x fish cue | 1 | 2.68 | 2.68 | 3.5 | 0.0859 | . |  |
| residuals | 12 | 9.2 | 0.77 |  |  |  |  |
| **EPA** | **df** | **SS** | **MS** | **F** | **p-value** |  |  |
| simulated DVM | 1 | 3.115 | 3.1148 | 8.648 | 0.0124 * |  |  |
| fish cue | 1 | 1.617 | 1.6168 | 4.489 | 0.0557 . |  |  |
| simulated DVM x fish cue | 1 | 2.803 | 2.8034 | 7.783 | 0.0163 * |  |  |
| residuals | 12 | 4.322 | 0.3602 |  |  |  |  |
| **ARA** | **df** | **SS** | **MS** | **F** | **p-value** |  |  |
| simulated DVM | 1 | 1.4004 | 1.4004 | 91.214 | **5.87E-07** | *** |  |
| fish cue | 1 | 0.1111 | 0.1111 | 7.235 | **0.01968** | * |  |
| simulated DVM x fish cue | 1 | 0.154 | 0.154 | 10.033 | **0.00811** | ** |  |
| residuals | 12 | 0.1842 | 0.0154 |  |  |  |  |
| **ALA** | **df** | **SS** | **MS** | **F** | **p-value** |  | **relative allocation** |
| simulated DVM | 1 | 25.34 | 25.336 | 4.688 | 0.0512 |  |  |
| fish cue | 1 | 9.48 | 9.482 | 1.754 | 0.21 |  |  |
| simulated DVM x fish cue | 1 | 2.81 | 2.811 | 0.52 | 0.4846 |  |  |
| residuals | 12 | 64.85 | 5.404 |  |  |  |  |
| **SDA** | **df** | **SS** | **MS** | **F** | **p-value** |  |  |
| simulated DVM | 1 | 41.16 | 41.16 | 13.232 | 0.0034 | ** |  |
| fish cue | 1 | 3.96 | 3.96 | 1.274 | 0.2811 |  |  |
| simulated DVM x fish cue | 1 | 3.03 | 3.03 | 0.976 | 0.3428 |  |  |
| residuals | 12 | 37.33 | 3.11 |  |  |  |  |
| **EPA** | **df** | **SS** | **MS** | **F** | **p-value** |  |  |
| simulated DVM | 1 | 87.98 | 87.98 | 14.21 | 0.00267 | ** |  |
| fish cue | 1 | 14.74 | 14.74 | 2.381 | 0.14878 |  |  |
| simulated DVM x fish cue | 1 | 1.42 | 1.42 | 0.229 | 0.64084 |  |  |
| residuals | 12 | 74.3 | 6.19 |  |  |  |  |
| **ARA** | **df** | **SS** | **MS** | **F** | **p-value** |  |  |
| simulated DVM | 1 | 9.48 | 9.481 | 1.526 | 0.24 |  |  |
| fish cue | 1 | 8.52 | 8.515 | 1.37 | 0.265 |  |  |
| simulated DVM x fish cue | 1 | 0.15 | 0.15 | 0.024 | 0.879 |  |  |
| residuals | 12 | 74.58 | 6.215 |  |  |  |  |
