# Supplementary material for: The impact of diel vertical migration on fatty acid patterns and allocation in Daphnia magna
Source: PeerJ. 2020 Apr 17;8:e8809. doi: 10.7717/peerj.8809 (PMC7169964; doi:10.7717/peerj.8809)
Supplement: Table S4 — Results of Two-Way ANOVAs on the effect of the factors ‘simulated DVM’ and ‘fish cue’ on the relative unsaturation index (UI) in Daphnia magna and their offspring. UI was calculated as the sum of the relative molar concentration of each fatty acid multiplied by its respective number of double bonds. Significant effects are highlighted in bold, N = 4. [file peerj-08-8809-s008.docx]

SI Table 6: Results of Two-Way ANOVAs on the effect of the factors ‘simulated DVM’ and ‘fish cue’ on the relative unsaturation index (UI) in *Daphnia magna* and their offspring.UI was calculated as the sum of the relative molar concentration of each fatty acid multiplied by its respective number of double bonds. Significant effects are highlighted in bold, N=4.

| **unsaturation index** | **Df** | **SS** | **MS** | **F** | **p-value** |  | **mothers** |
| --- | --- | --- | --- | --- | --- | --- | --- |
| simulated DVM | 1 | 0.028002 | 0.028002 | 17.433 | **0.00129** | ** |  |
| fish cue | 1 | 0.001495 | 0.001495 | 0.931 | 0.35367 |  |  |
| simulated DVM x fish cue | 1 | 0.000873 | 0.000873 | 0.543 | 0.47521 |  |  |
| residuals | 12 | 0.019274 | 0.001606 |  |  |  |  |
| **unsaturation index** | **Df** | **SS** | **MS** | **F** | **p-value** |  | **offspring** |
| simulated DVM | 1 | 0.18069 0 | 0.18069 | 62.616 | **4.2e-06** | *** |  |
| fish cue | 1 | 0.00028 0 | 0.00028 | 0.096 | 0.762 |  |  |
| simulated DVM x fish cue | 1 | 0.00015 0 | 0.00015 | 0.051 | 0.825 |  |  |
| residuals | 12 | 0.03463 0 | 0.00289 |  |  |  |  |
